# Does ultrasound agree with parent's perception of joint disease in juvenile idiopathic arthritis?

**DOI:** 10.1186/1546-0096-12-S1-P172

**Published:** 2014-09-17

**Authors:** Carlo Biancardi, Irene Borzani, Sofia Torreggiani, Antonella Petaccia, Angelo Ravelli, Fabrizia Corona, Giovanni Filocamo

**Affiliations:** 1 UOS Reumatologia Pediatria, Dipartimento della Donna, del Bambino e del Neonato, Milano, Italy; 2 UO Radiologia Pediatrica, Dipartimento della Donna, del Bambino e del Neonato, Fondazione IRCCS Cà Granda Ospedale Maggiore Policlinico, Milano, Italy; 3 Pediatria II, Istituto Giannina Gaslini and Università di Genova, Genoa, Italy

## Introduction

Ultrasound (US) is a powerful tool for the assessment of joint disease in children with juvenile idiopathic arthritis (JIA) and has been shown to be more accurate than clinical examination in detecting synovitis. Parent’s proxy-report of joint involvement is potentially usefulto obtain information on parent’s perception of the burden of child’s arthritis and may serve as surrogate for physician’s articular examination. However, it is unclear whether parents are reliable reporters of their children’s disease.

## Objectives

To evaluate the level of agreement between parents’ proxy-report of joint involvement and US assessment of joint synovitis in children with JIA.

## Methods

Before the study visit, parents of children with JIA were asked to complete the Juvenile Arthritis Multidimensional Assessment Report (JAMAR), which includes a standardized assessment of the presence of swelling or pain in 9 joints or joint groups, and several other parent-centered JIA outcome measures. At study visit, a pediatric rheumatologist, who was unaware of parent’s reports, performed a formal joint assessment and scored the presence or absence of swelling and tenderness/pain on motion in the same joints assessed by the parent. After the visit, a pediatric radiologist with more then 5 years of experience in US assessment in JIA evaluated independentlythe presence of synovial hypertrophy/effusion (gray scale US - GSUS) and Power Doppler (PD) inmetacarpophalangeal and interphalangeal joints, knees and ankles, and quantified each US featureon a 0-3 semi-quantitative scale. Agreement between parent, rheumatologist, and ultrasonographerin joint assessment was computed by means of Cohen’s kappa and was categorized as follows:<0.40=poor; 0.41-0.60=moderate; 0.61-0.80=substantial; >0.80 excellent.

## Results

The JAMAR was completed by parents of 10 unselected patients, 8 with persistent oligoarthritis, 1 with extended oligoarthritis and 1 with rheumatoid factor-negative polyarthritis, aged 22 months to 8 years. The median (range) of JADAS71 in the 10 patients was 10 (0-19).

Table 1 shows the k values for agreement in joint assessment between parents, physician and ultrasonographer evaluation.

**Table 1:** 

Joint	GSUS vs parent	PDUS vs parent	GSUS vs physician swelling	PDUS vs physician swelling	GSUS vs physician pain/LOM	PDUS vs physician pain/LOM
Hand joints	0.62	-£	0.62	-£	0.62	-£

Knee	0.70	0.67	0.62	0.77	0.62	0.77

Ankle	0.47	0.62	0.57	0.69	0.47	0.62

£Not assessed because PDUS score was = 0.

## Conclusion

Our results show moderate-to-substantial agreement between parents’ proxy report of joint disease and US assessment. Concordance with US was similar for parents and physicians. This finding suggests that parents are reliable reporters of the extension and severity of their children’s arthritis. Overall, concordance was greater for PDUS than for GSUS and was lower for the ankle than for the other joints.

## Disclosure of interest

None declared.

